# Production of Cu_0.5_Zn_0.5_Fe_2_O_4_ Nanostructures as a Hyperthermia Agent for Cancer Healing

**DOI:** 10.1155/ijbm/7290633

**Published:** 2025-05-15

**Authors:** Hashim Hamood Jabbar Al-Gburi, Sayed Ali Hassanzadeh-Tabrizi, Saeid Jabbarzare

**Affiliations:** Institute of Manufacturing Engineering and Industrial Technologies, Na.C, Islamic Azad University, Najafabad, Iran

**Keywords:** hyperthermia, magnetic properties, nanostructures, sol-gel combustion method, spinel ferrite

## Abstract

Cancer is a pervasive and devastating disease affecting various parts of the body, posing significant challenges to human societies. Recently, the development of novel magnetic and biocompatible nanoparticles has emerged as a promising approach for magnetic hyperthermia in cancer treatment, complementing existing therapeutic methods. In the present work, Cu_0.5_Zn_0.5_Fe_2_O_4_ mixed spinel nanoparticles were produced via a sol-gel combustion route. The produced magnetic nanopowders were studied via FTIR, SEM, XRD, and VSM techniques. XRD results confirmed the formation of the spinel structure of ferrites. Microstructural investigations showed that the synthesized nanoparticles have a particle size ranging from 20 to 200 nm. The VSM results displayed that the saturation magnetization and coercivity of Cu_0.5_Zn_0.5_Fe_2_O_4_ nanoparticles were 57 emu/g and 24 Oe, respectively. Saturation magnetization for the Cu_0.5_Zn_0.5_Fe_2_O_4_ specimens improved with increasing heat treatment temperature. In order to examine the samples' heating effectiveness for magnetic hyperthermia therapy, various magnetic fields were used. The temperature of the Cu_0.5_Zn_0.5_Fe_2_O_4_ powders increased from 37°C to 47°C in 10 min when exposed to a 400-Oe magnetic field and 200-kHz frequency. Results showed that the fabricated products have the potential to be used as hyperthermia agents for cancer therapy. The novelty of this study focuses on the use of Cu_0.5_Zn_0.5_Fe_2_O_4_ mixed spinel as a new hyperthermia agent with more biocompatible constituent elements.

## 1. Introduction

Magnetic nanoparticles have recently demonstrated significant promise for use in several fields, including magnetic separation [1], supercapacitors [2], photocatalysts [3], drug administration [4], magnetic imaging [5], magnetoelectronic [6], and cancer treatments involving hyperthermia [7–9]. Specifically, cancer treatment is possible by magnetic nanoparticles to undergo hyperthermia, which is the process by which they transform lost magnetic energy into heat. The process for this type of hyperthermia involves heating the cancer-affected area to 43°C–45°C, which is accomplished with magnetic materials in a magnetic field with high frequency. This process can kill cancer cells with the least impact on healthy cells, suggesting that it may be utilized in targeted, cost-effective, and side-effect-free treatments [10–12].

Among different magnetic materials, spinel ferrites have gained much attention in recent years because of their exceptional characteristics. Spinel ferrites exhibit strong magnetic properties, including high saturation magnetization, low coercivity, and high permeability [13]. In addition, they have high electrical resistivity, which reduces eddy current losses in magnetic applications. This property is particularly useful in high-frequency applications [14]. These kinds of magnetic materials are chemically stable and resistant to corrosion, which enhances their durability and longevity in various environments, especially in the corrosive environment of the body [15]. This property makes them more biocompatible compared to metallic magnetic materials. They have a general formula of NFe_2_O_4_. In this formula, N is a divalent metal ion. Sometimes, different divalent metal ions were used simultaneously to improve the magnetic properties of the fabricated spinel ferrites. Spinel ferrites have a wide range of uses, such as pigments, sensors, hyperthermia agents for cancer therapy, magnetic recording media, magnetic fluids for information storage, magnetic resonance imaging (MRI) contrast agents, and photocatalysts [16, 17]. It was reported that the substitution in the spinel structure with different elements in a controlled manner is useful for the improvement of the magnetic properties [18].

Nano spinel ferrites have been created using various synthesis techniques. Most of the fabrication techniques documented in the literature are co-precipitation [19], sol-gel [20], Pechini approach [21], solvothermal [22], polyacrylamide gel [23], mechanical milling [24], and hydrothermal [25]. Each one of these methods has its pros and cons. Some of them require a lot of energy and time to create nanoparticles. Furthermore, some of them need the use of costly, dangerous, and poisonous solvents. Sol-gel combustion is one of these techniques that has a lot of benefits. Reactions can be accomplished quickly with this strategy. In addition, high processing temperatures can be achieved with this method, which facilitates the production reactions. Moreover, it produces a homogeneous, high-purity material with nanoparticle sizes and permits superior stoichiometric control. Therefore, it seems the sol-gel combustion method is a suitable process for creating complex ceramic oxide materials [20, 26, 27].

The use of simple ferrites such as Fe_3_O_4_ and ferrites containing elements like NiFe_2_O_4_ or CoFe_2_O_4_ demonstrated cytotoxic effects due to the release of ions in concentrations higher than those tolerated by cells. Therefore, spinel ferrites containing multiple and more beneficial elements can be a suitable choice to mitigate this detrimental effect. Zinc is a vital element for the development and function of immune cells [28] and is involved in every stage of the wound healing process [29]. In addition, this element is required for the catalytic activity of hundreds of enzymes involved in protein synthesis [30]. Copper also helps wound healing [31]. Copper plays a role in the immune system by helping to fight off infections and supporting the production of white blood cells [32]. Based on the above discussion, it can be concluded that a mixed spinel containing copper and zinc could be a good choice for medical use.

To the best of our knowledge, a few studies are working on zinc–copper ferrite as a hyperthermia agent. Thus, in the present work, Cu_0.5_Zn_0.5_Fe_2_O_4_ nanoparticles were created in the current study using a sol-gel combustion method. The potential of Cu_0.5_Zn_0.5_Fe_2_O_4_ spinel ferrites for hyperthermia treatment was also evaluated in vitro.

## 2. Experimental Procedure

### 2.1. Materials and Methods

**Figure 1:**
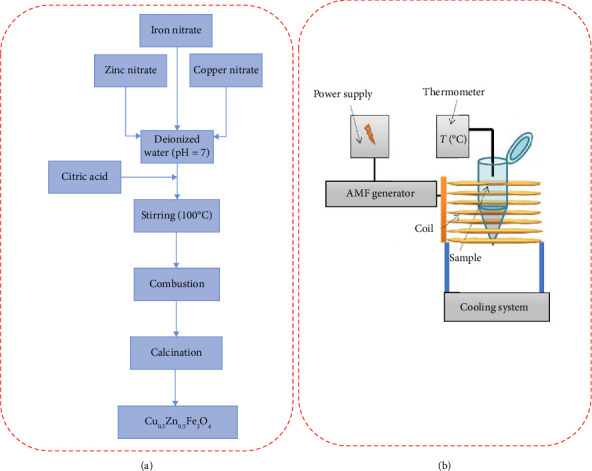
Schematic illustration of the (a) fabrication process and (b) hyperthermia setup.

Citric acid (C_6_H_8_O_7_) (99.5%, Merck), Zn(NO_3_)_2_·6H_2_O (≥ 98%, Merck), Cu(NO_3_)_2_·6H_2_O (99.5%, Merck), and Fe(NO_3_)_3_·9H_2_O ((≥ 99%, Merck) were used for the preparation of Cu_0.5_Zn_0.5_Fe_2_O_4_ samples. Sol-gel combustion was employed to create magnetic nanostructures. First, metal salts were dissolved in deionized water under magnetic stirring (100 rpm) at 80°C. For this aim, 2.51 g of copper nitrate, 16.79 g of iron nitrate, and 3.1 g of zinc nitrate were dissolved sequentially in 100 mL of distilled water. The molar ratio of Fe:Cu:Zn was 2:0.5:0.5. Then, 11.52 g of citric acid was added to the solution. The metal salt to the citric acid molar ratio was equal to 1. An ammonium hydroxide solution (25%wt.) was added to adjust the pH to 7. The prepared solution was heated at 200°C until its water evaporated. There was a self-combustion reaction that produced a black powder. The powders were then crashed and calcined at 800°C.  Figure 1(a) provides a schematic illustration of the synthesis process.

### 2.2. Characterization

The crystal structure of the Cu_0.5_Zn_0.5_Fe_2_O_4_ specimens was identified using X-ray diffraction analysis (Phillips PW-1710 equipment, Cu-Kα irradiation at *λ* = 1.54059 Å) at 30 mA current and 40 kV voltage with a scanning speed of 2°/min. Using a Fourier transform infrared (FTIR, JASCO6300), the chemical bonding of the synthetic spinel ferrites was examined. A scanning electron microscope (SEM, TESCAN) was used to record the microstructural analyses of Cu_0.5_Zn_0.5_Fe_2_O_4_ samples. The magnetic characteristics of the synthesized spinel ferrites were assessed using a vibrating sample magnetometer (VSM, HH-15). A homemade induction machine with a 2-cm radius and an 8-turn coil was used to gauge spinel ferrites' heating efficiency. The sample's temperature changes within the designated time after the application of a certain magnetic field were monitored using a thermometer.

### 2.3. Hyperthermia Test of Samples

The heating efficiency of spinel ferrites was measured using a homemade induction machine with an 8-turn coil and a 2-cm radius. The schematic illustration of the used setup is shown in Figure 1(b). This was accomplished by placing an insulated tube at the center of an induction coil that contained 1 mL of magnetic fluid. The spinel ferrite concentration of this fluid was 15 mg/mL. After applying a particular magnetic field for the designated amount of time, the temperature variations of samples were recorded using a thermometer.

### 2.4. Cell Culture and MTT Assay

The MG63 cell line (Pasteur Institute) was used to evaluate the samples' compatibility in vitro. After that, the cells were put in a flask and kept there until they reached 80% confluency. The dimethylthiazol diphenyltetrazolium bromide compound (MTT, Sigma-Aldrich) was used to assess cell compatibility. A 96-well microtiter plate was then filled with 10^3^ cells per well. The sterilized samples were then added to the culture media (500 mg/mL). The obtained extracts were put into the cell plates. Sterile PBS was mixed with 0.5 mg/mL of MTT for the cytotoxicity test. Following the separation of the culture media and culture cells, the cells were treated with 500 L of MTT solution and placed in a CO_2_ incubator set at 37°C for 4 h. After dissolving the formazan crystals that had developed in each well with 350 μL of DMSO solution, the wells were incubated for two more hours. Lastly, an ELISA microplate reader was used to measure the optical density of 200 μL of the supernatant at 570 nm.

## 3. Results and Discussion

**Figure 2:**
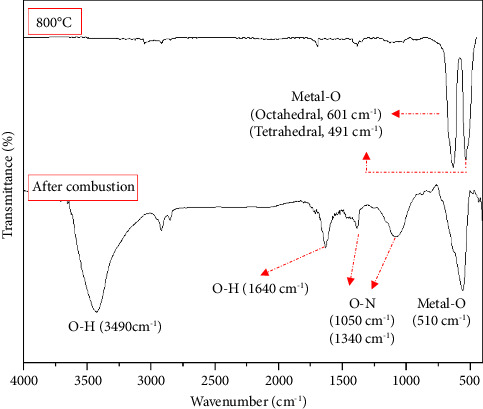
FTIR of the samples after combustion and calcination at 800°C.

Figure 2 shows the FTIR results of the samples after combustion and after calcination at 800°C. The stretching and bending vibrations of O-H groups may be responsible for the large absorption peaks at about 3490 and 1645 cm^−1^, which indicate the presence of water in the prepared samples [33]. After calcination, these peaks disappear, which shows the removal of water from the samples. Nitrate groups are relevant to the absorption peak at around 1050 and 1340 cm^−1^ [34, 35]. The nitride metal salts that are used as raw materials are the source of these nitrate groups. Nitrate groups broke down during calcination, and hence, this peak was eliminated from the FTIR analysis. The broad absorption peak around 510 cm^−1^ is related to metal-oxygen vibration. However, after the calcination process, this broad peak turns into two sharp peaks that show rearrangement of the structure. The peaks at about 601 and 491 cm^−1^ are pertinent to metal-O vibrations at octahedral and tetrahedral positions, respectively, indicating the development of the cubic spinel ferrite structure [36, 37].

**Figure 3:**
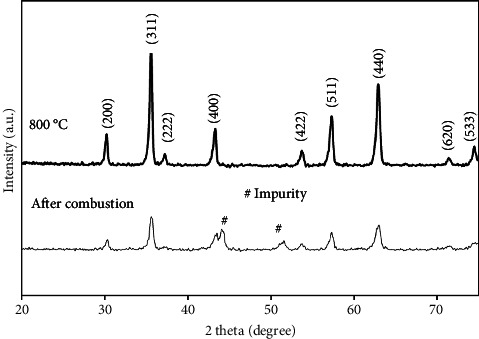
XRD of the samples after combustion and calcination at 800°C.

The XRD patterns of the specimens that were heat-treated at different temperatures are shown in Figure 3. In the prepared sample, the peaks at 30.2°, 35.5°, 37.1°, 43.2°, 53.5°, 57.1°, 62.8°, 71.1°, and 74.1° pertain to (220), (311), (222), (400), (422), (511), (440), (620), and (533) crystal planes, respectively, which confirm the formation of cubic spinel ferrite structure with Fd-3m space group (JCPDS card no. 01-077-0012). In addition, peaks associated with impurities like Fe_2_O_3_, indicating the produced heat during combustion has not been high enough to produce single-phase spinel ferrite. In the combustion process, the solution boiled, foamed, ignited, and burned as a glowing flame, producing the fluffy, voluminous solids. In this method, the fuel (citric acid) and oxidizer (hydrated metal nitrates) create an exothermic reaction. It should be mentioned that the fuel is not only to produce heat for the reactions but also to produce complexes with the metal ions to improve their solubility and inhibit the precipitation of the metal ions. Many gaseous byproducts are produced during the sol-gel combustion process. The solid product expands significantly due to this gasification, and the temperature rapidly drops following the reaction, making the solid product porous and finely disseminated. These characteristics cause nanoscale powders to be produced in this method [38]. However, rapid cooling of the powder may hinder complete reactions between raw materials to form a single-phase spinel ferrite structure. The peaks related to impurities vanished during the calcination at 800°C, and spinel ferrite-related peaks were just seen, indicating that the raw materials had reacted to produce the final product. The crystallite size (D) of the samples was measured based on the following equation [39]:

(1) $$\begin{array}{c} D=\frac{K\lambda }{\beta \;\cos\;\theta }, \end{array}$$

where the Bragg angle is *θ*, the full width at half maximum is β, the X-ray wavelength is *λ*, and *k* is a constant. D was determined to be 21 and 68 nm for the samples after combustion and calcination at 800°C, respectively. A higher temperature during calcination promotes diffusion mechanisms inside the spinel ferrite structure, hence expediting the development of crystallites. The lattice strain (ε) was calculated by equation (2) [40]. Near a defect such as a dislocation, the crystal lattice is elastically strained or bent. The diffraction peaks broaden as a result of such elastic fields. This effect, which is dependent on nonuniform lattice distortions, is sometimes referred to as microstrain [41]:

(2) $$\begin{array}{c} \varepsilon =\frac{\beta }{4\;\tan\;\theta }. \end{array}$$

The lattice strain is found to be 2.99 × 10^−3^ and 0.922 × 10^−3^ for the samples after combustion and calcination at 800°C, respectively. As can be seen with an increase in the calcination temperature microstrain reduces. It can be attributed to the stress relief of spinel structure due to the diffusion of atoms during the growth of particles.

The lattice constant (α) of the specimens can be calculated by the following equation [42]:

(3) $$\begin{array}{c} a=\lambda \frac{\sqrt{h^{2}+k^{2}+l^{2}}}{2\;\sin\;\theta }, \end{array}$$

where *θ* is the diffraction angle and (h k l) are Miller indices corresponding to crystal planes. However, for precise calculation of the lattice parameter, the corrected lattice parameter was estimated by the Nelson–Riley method (equation (4)):

(4) $$\begin{array}{c} f\left(\theta \right)=\frac{1}{2}\left(\frac{\cos^{2}\;\theta }{\sin\;\theta }+\frac{\cos^{2}\;\theta }{\theta }\right). \end{array}$$

**Figure 4:**
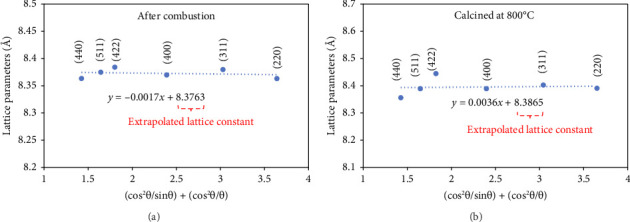
Nelson–Riley plots for calculation of extrapolated lattice parameters of Cu_0.5_Zn_0.5_Fe_2_O_4_ samples after combustion and calcination at 800°C.

This method involves plotting the lattice parameter values obtained from diffraction peaks via equation (3) against a function of the diffraction angle. For this aim, by plotting the lattice parameter values against *F*(*θ*), it can be possible to extrapolate *F*(*θ*) = 0 to obtain a more accurate estimate of the true lattice parameter. This method helps to minimize systematic errors that may arise from factors such as instrumental imperfections or sample misalignment [43, 44].  Figure 4 represents the Nelson–Riley plot for the samples after combustion and after calcination at 800°C. The calculated lattice constant increased from 8.376 to 8.386 Å after calcination which is closer to theoretical value of this structure (8.388 Å) according to JCPDS file no. 01-077-0012. The better agreement of lattice parameter of calcined sample with theoretical value could be attributed to crystal structure refinement during calcination.

**Figure 5:**
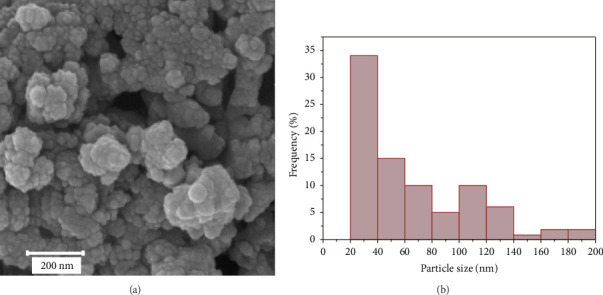
(a) SEM image and (b) particle size histogram of the Cu_0.5_Zn_0.5_Fe_2_O_4_ after calcination at 800°C.

The SEM image of the Cu_0.5_Zn_0.5_Fe_2_O_4_ sample is displayed in Figure 5(a). In the powders, agglomerates of various sizes are seen. In addition, some holes can be seen in the structure. As is well known, the combustion process generates a lot of gas, which leaves the products with numerous holes during removal.  Figure 5(b) shows the particle size distribution histogram obtained from the SEM image. The histogram displays a broad variation of particle sizes, ranging from 20 to 200 nm. This wide range of sizes is due to the formation of agglomeration of the powders. Aggregated nanoparticle formation is a common occurrence in powders with nanosized particles. Nanoparticles have high surface energy because of their huge surface area. Therefore, particles attach to each other to reduce their surface energy [45]. It was reported that the particle size distribution between 20 and 200 nm is optimum to use as injectable biomaterial [46]. Nanoparticles larger than 200 nm are removed from the body due to macrophagic uptake, while nanoparticles smaller than 20 nm have a low circulation lifetime because of kidney function.

**Figure 6:**
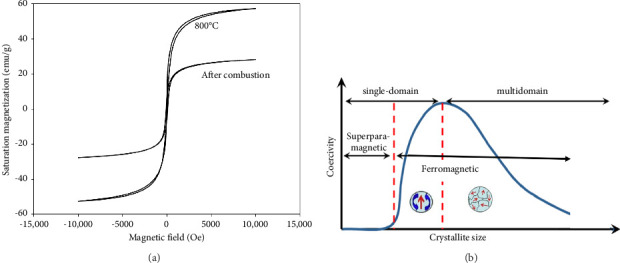
(a) Magnetic properties of the Cu_0.5_Zn_0.5_Fe_2_O_4_ samples and (b) the qualitative characteristic of the size-dependent coercivity of spinel ferrites.

The magnetic characteristics of the Cu_0.5_Zn_0.5_Fe_2_O_4_ samples are depicted in Figure 6(a). As can be seen, samples show ferrimagnetic behavior. The low coercivity (Hc) of calcined specimens leads to the classification of these materials as soft magnets. As the calcination temperature rose, the saturation magnetization (Ms) increased. According to the VSM data, the untreated sample had a saturation magnetization of 35 emu/g and a coercivity of 80 Oe. However, Cu_0.5_Zn_0.5_Fe_2_O_4_ nanoparticles calcined at 800°C had a saturation magnetization of 57 emu/g and a coercivity of 21 Oe. As can be seen with increasing calcination temperature, saturation magnetization increases, whereas coercivity reduces. Higher calcination temperatures have the potential to produce larger crystallites, increased product crystallization, and an enhancement of the super-exchange interaction between the components. Furthermore, an additional explanation for the rise in Ms with increasing calcination temperature could be a decrease in porosity. When the porosity decreases, the particles get closer together and more magnetic moments align, potentially increasing Ms [47, 48].

Reduction in coercivity with an increase in calcination temperature could be explained by typical size-dependent coercivity of spinel ferrites as shown in Figure 6(b). According to this theory, as particle size decreases, coercivity increases until it reaches a maximum value at the critical diameter, which is the state where the multidomain transitions to the single-domain occur. In such a situation, the highest coercivity is obtained. When particle size reduces below this critical size, the amount of coercivity decreases to reach zero. This behavior is known as superparamagnetic [49]. Based on the obtained results in the present study and the above discussion, it can be concluded that the produced nanoparticles are multidomain because increasing the size after calcination resulted in a reduction in coercivity. Furthermore, it was stated that coercivity can be impacted by porosity. A higher coercivity is the result of domain walls that are unable to move freely and must align with a strong magnetic field when pores are present [50]. In multidomain nanoparticles, the relation between coercivity and particle size (D) is defined by the following equation [51]:

(5) $$\begin{array}{c} \text{Coercivity}=a+\frac{b}{D}, \end{array}$$

where *a* and *b* are constants. According to equation (3), an increase in particle size causes a reduction in coercivity. As it is known in multidomain magnetic materials, the rotation of magnetic domains results in a magnetic ferrite becoming magnetized or demagnetized. In such cases, drain boundaries tend to resist this domain rotation. With an increase in crystallite size, there are fewer grain boundaries present, leading to decreased energy requirements for the magnetization or demagnetization of the domains. As a result, this implies a lower coercivity for the spinel ferrite.

An essential magnetic property for spinel ferrite nanomaterials is the anisotropy constant or anisotropy energy density (*K*_eff_). This factor influences the Néel relaxation time, which is crucial for various applications. In magnetic storage mediums, it is desirable to have a high *K*_eff_, while in magnetic imaging, a small *K*_eff_ is preferred. The calculation of *K*_eff_ can be done using the following equation [52, 53]:

(6) $$\begin{array}{c} k_{\text{eff}}=\frac{M_{s}\times H_{c}}{0.985}. \end{array}$$

*K*_eff_ for untreated and calcined samples was calculated to be 2842.6 and 1108.6 erg/g, respectively. It is important to note that the anisotropy constant is mainly influenced by the substance's chemical structure. Moreover, factors like particle shape, internal stress, and surface effects also play an important role [54, 55].

Squareness in magnetic properties is defined as the ratio of the remanent magnetization to the saturation magnetization (Mr/Ms). High squareness indicates that the material retains a significant amount of its magnetization after removing the external field, which is desirable for permanent magnets and magnetic storage applications. Low squareness shows that the material loses most of its magnetization when the external field is removed, which is typical for soft magnetic materials used in transformers and inductors. Squareness for samples after combustion and calcination at 800°C was 0.17 and 0.06, respectively. In the context of magnetic hyperthermia and medical applications, a low squareness ratio is generally preferred because magnetic nanoparticles do not retain magnetization in the absence of an external magnetic field. This property is crucial for minimizing potential side effects and ensuring that the particles do not aggregate, increasing their biocompatibility [56–58].

**Figure 7:**
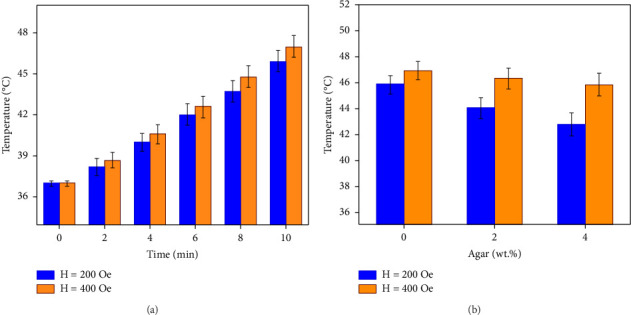
Heat generation of the Cu_0.5_Zn_0.5_Fe_2_O_4_ specimens calcined at 800°C with 200 and 400 Oe field amplitudes and frequency of 200 kHz (a) at different times and (b) at different agar concentrations.

The heat generation ability of the magnetic Cu_0.5_Zn_0.5_Fe_2_O_4_ spinel ferrites calcined at 800°C was evaluated using different magnetic fields of 200 and 400 Oe at a frequency of 200 kHz. The findings are displayed in Figure 7(a). It is discovered that the temperature rises with increasing time after applying the AC field. Moreover, the temperature rises with increasing the field amplitude. Thus, the treatment regime for treating hyperthermia can be achieved by varying the magnitude and duration of the magnetic field. It is observed that the curves for both magnetic fields of 200 and 400 Oe reach the requisite range, which is the prerequisite for hyperthermia (42°C–45°C [59]). The heat-producing properties of magnetic nanoparticles dispersed in a liquid medium could be attributed to four different mechanisms. They include Brownian loss, Néel relaxation, hysteresis loss, and eddy current [60, 61]. Relatively little eddy current loss occurs in the generated nanoparticles because Cu_0.5_Zn_0.5_Fe_2_O_4_ is an insulator. Magnetic properties of the samples showed that the hysteresis loss of samples is low which confirms this mechanism is not prominent for heating generation. Thus, the active mechanisms in this system might be Brownian loss and Néel loss. A direction change in the magnetic moment is the main factor responsible for heat generation via Néel relaxation. The physical rotation of magnetic nanoparticles in a liquid medium produces the final mechanism of heat generation, referred to as Brownian relaxation. Somvanshi et al. [62] synthesized Zn-Mg ferrite nanoparticles for magnetic hyperthermia and reported that higher concentrations of samples produce higher temperatures for hyperthermic treatment.

It is crucial to recognize that in vivo and in vitro magnetic hyperthermia tests may yield different results. Specifically, the viscosity of media and type of the tissue in which spinel ferrites are dispersed influence the amount of heat produced. For example, the physical rotation of spinel ferrites is restricted in the body environment due to the higher viscosity of cancer cells. To investigate the effect of the viscosity on hyperthermia efficiency, calcined specimens were placed in a viscous mixture of water and agar.  Figure 7(b) illustrates the heating capacity of the specimens across different media. It is clear that heat generation decreases as viscosity increases, highlighting the significance of Brownian relaxation in heat generation. The heating generation capacity difference of the specimens in the water and 4 wt.% agar solution is approximately 6.7% at 200 Oe magnetic field, but this difference diminishes to 2.4% at a magnetic field of 400 Oe.

**Figure 8:**
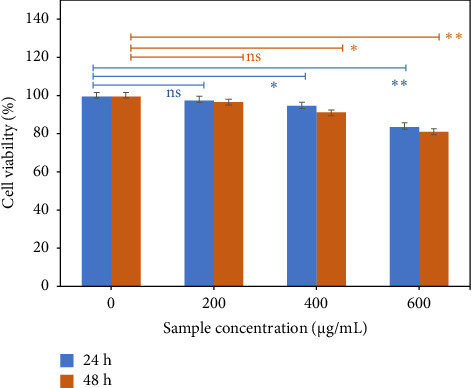
Viability of the MG63 cells incubated for 24 and 48 h with extracted medium containing different concentrations of the Cu_0.5_Zn_0.5_Fe_2_O_4_ specimens calcined at 800°C (The tests were carried out with 3 replicates, and the results were given as mean ± standard deviation (ns = not significant, ^∗^*p* ≤ 0.05, ^∗∗^*p* ≤ 0.01).

Three important factors should be taken into account when magnetic nanoparticles are supposed to be used as hyperthermia agents. First is the safety limit of nanoparticles in cytotoxicity concepts. MTT assay was employed to investigate the cytotoxicity of calcined Cu_0.5_Zn_0.5_Fe_2_O_4_ spinel nanoparticles. The viabilities of the MG63 cell cultured in extracted media for 24 h, containing varying concentrations of the spinel ferrite samples, are displayed in Figure 8. As the amount of nanopowder increases, cell viability decreases. Magnetic ferrite nanopowders have a hazardous effect because they release ions into the water, which can damage cells if the concentrations are higher than what the cells can withstand [63–65]. Additionally, another way that magnetic ferrite causes cytotoxicity is by the production of reactive oxygen species [66]. However, the cell viability of all samples is above 70%, which is acceptable for medical use. These results confirm that the produced nanoparticles are biocompatible but should be used in proper concentration.

Second, magnetic field safety is also an important factor in hyperthermia medical treatment. The Brezovich principle defines a safety limit where the *f* × *H* value should not be larger than 5 × 10^8^ A/ms (∼6 × 10^6^ Oe/s) according to medical tolerance findings carried out on healthy individuals. When it comes to body parts, it has been observed that this stringent limitation can be exceeded up to 10 times [67, 68]. The *H* × *f* value in this study is 8 × 10^4^ Oe/s, which falls within the range for usage in clinical hyperthermia.

The third key requirement for hyperthermia treatment with magnetic nanoparticles is a proper specific absorption rate (SAR). SAR quantifies the power of heat generation per unit mass of materials, enabling the use of lower magnetic field strengths and frequencies, as well as reducing the amount of nanoparticles needed for effective hyperthermia treatment. For materials with lower SAR, larger quantities should be utilized, which poses a challenge due to the limited ability of cells to absorb nanoparticles without cytotoxicity. To quantify the hyperthermia, equation (7) was employed to measure the SAR in watts per gram of material [69]. When an alternating current (AC) magnetic field is applied, the SAR value reflects the heating capability of the magnetic particles. A higher SAR indicates greater heating efficiency at lower nanoparticle dosages, thereby enhancing the management of hyperthermia:

(7) $$\begin{array}{c} \text{SAR}=C_{p}\frac{M_{s}}{M_{n}}\left(\frac{dT}{dt}\right). \end{array}$$

Here, *M*_*n*_ and *M*_*s*_ denote the mass of nanoparticles and the suspension, respectively. *C*_*p*_ represents the specific heat capacity of the suspension. The term dT/dt signifies the initial slope of the heating curve. The results were obtained at 200-kHz frequency and 200- and 400-Oe magnetic fields. The SAR value for calcined Cu_0.5_Zn_0.5_Fe_2_O_4_ spinel nanoparticles was 46.2 and 51.4 W/g at 200- and 400-Oe magnetic fields, respectively. As can be seen, the SAR of the samples increases with a stronger magnetic field. As mentioned before, higher SAR is known to be beneficial for treating hyperthermia because it increases heating efficiency with a lower dosage of magnetic nanoparticles, reducing the likelihood of cytotoxicity and an immunological reaction. It seems that a higher magnetic field increases the function of heating mechanisms. It was reported that several factors affect SAR value. Intrinsic factors like higher magnetization and magnetocrystalline anisotropy energy can cause an increase in SAR [70]. Extrinsic factors such as nanoparticle concentration and dispersity also have an important effect on SAR. For instance, increasing the nanoparticle concentration causes changes in Néel's relaxation time and, consequently, a reduction in SAR value [71]. Bohara et al. [72] reported that high particle concentrations may cause particle agglomeration, which in turn may intensify the dipolar contact between the particles and reduce power dissipation to the medium. In addition, enhancement in the dispersing of magnetic nanoparticles can increase SAR [73]. Mohana and Sumathi [74] reported that the method of synthesis can change SAR values.

## 4. Conclusion

Cu_0.5_Zn_0.5_Fe_2_O_4_ cubic spinel magnetic nanoparticles were created using the sol-gel combustion process. The effects of heat treatment on the magnetic characteristics and structure of the produced spinel ferrites were studied. XRD results confirmed the formation of cubic spinel ferrites. The saturation magnetization of the samples was enhanced by heat treatment. Cu_0.5_Zn_0.5_Fe_2_O_4_ nanoparticles calcined at 800°C showed a coercivity of 24 Oe and a saturation magnetization of 57 emu/g, based on the VSM results. The SEM micrograph shows a wide range of particle sizes, from 20 to 200 nm. The heating production ability of the Cu_0.5_Zn_0.5_Fe_2_O_4_ nanoparticles calcined at 800°C showed that they can produce proper thermal energy for hyperthermia treatment under AC magnetic fields of 200 and 400 Oe at a frequency of 200 kHz. The MTT assay confirmed the biocompatible nature of the Cu_0.5_Zn_0.5_Fe_2_O_4_ nanoparticles. However, in high concentrations of nanoparticles, cytotoxic effects were observed. These results indicate that the synthesized magnetic ferrites are promising candidates for magnetic hyperthermia in cancer treatments. Surface modifications for obtaining higher biocompatibility may be a possible future research direction.

## Data Availability

The data that support the findings of this study are available from the corresponding author upon reasonable request.
